# Suprasellar Epidermoid Cyst Originating from the Infundibulum: Case Report and Literature Review

**DOI:** 10.7759/cureus.3226

**Published:** 2018-08-29

**Authors:** Erin P McCormack, Justin M Cappuzzo, Zachary Litvack, M. Isabel Almira-Suarez, Jonathan S Sherman

**Affiliations:** 1 Surgery, Ochsner Medical Center, Jefferson, USA; 2 Neurosurgery, University at Buffalo/State University of New York, Buffalo, USA; 3 Neurological Surgery, Swedish Brain and Spine Specialists, Seattle, USA; 4 Neuropathology, Children's National Medical Center, Washington, DC, USA; 5 Neurological Surgery, George Washington University, Washington DC, USA

**Keywords:** epidermoid cyst, pituitary, stalk, infundibulum

## Abstract

Epidermoid cysts account for a small fraction of intracranial brain tumors, most commonly found in the cerebellopontine angle and parasellar cisterns. Here we present a rare case of an epidermoid cyst located in the suprasellar region, specifically originating from the infundibulum. Only one additional case with an epidermoid cyst originating within the pituitary stalk has been previously reported in the literature. The patient in this case presented with headaches, diplopia and blurred vision without any endocrinopathy. The patient’s pre-operative evaluation was significant for pseudotumor cerebri, hyponatremia, obesity, and a history of smoking; post-operative course was significant for neurogenic diabetes insipidus.

## Introduction

Epidermoid cysts account for approximately 1%-2% of all brain tumors and are most commonly found in the cerebellopontine angle and parasellar cisterns [1]. The slow growth of these tumors often results in them remaining asymptomatic until their size is large enough to compress surrounding structures, such as the pituitary stalk or optic chiasm [2]. Most often these tumors are diagnosed in adults aged 20 to 40 years old, with incidence peaking in the fourth decade of life [3]. A transsphenoidal approach to the removal of these tumors has been shown to reduce morbidity and mortality in these patients due to the better visualization of the neoplasm and surrounding anatomy and minimal (if any) brain retraction [2].

Tumors and cysts of the pituitary stalk and hypothalamic region vary in presentation depending on their location, progression, and extension into the surrounding anatomy, in addition to the age and comorbidities of the patient; all of these factors must be addressed prior to surgery [4].

## Case presentation

History

A 36-year-old female presented to the Emergency Department (ED) with a persistent generalized headache, dizziness, and blurred vision in addition to diplopia. On physical exam, she had normal range of motion and strength in all extremities and no focal neurological deficit was observed other than a lagging of her left eye while testing her extraocular muscles. The patient was discharged from the ED with instruction to follow up with the neurosurgery department as an outpatient.

At her outpatient neurosurgery appointment, the patient revealed that her headache had increased in severity and had begun to localize to the bridge of her nose and medial forehead. Her headaches were accompanied by diplopia, photophobia, blurred vision, nausea, and vomiting. Of note, the patient’s past medical history was significant for obesity, smoking, and benign essential hypertension controlled with medication. Her neuro-ophthalmologic exam was notable for enlarged blind spots bilaterally with possible inferonasal and temporal field defects. Ophthalmology also noted 20/30 vision with decreased colour vision, an afferent papillary defect in the right eye, and hemorrhagic disc elevation in both eyes with the hemorrhage extending into the macula of her right eye. Ophthalmology’s recommendation was urgent decompression of the optic chiasm, which was in agreement with neurosurgery’s recommendation.

**Figure 1:**
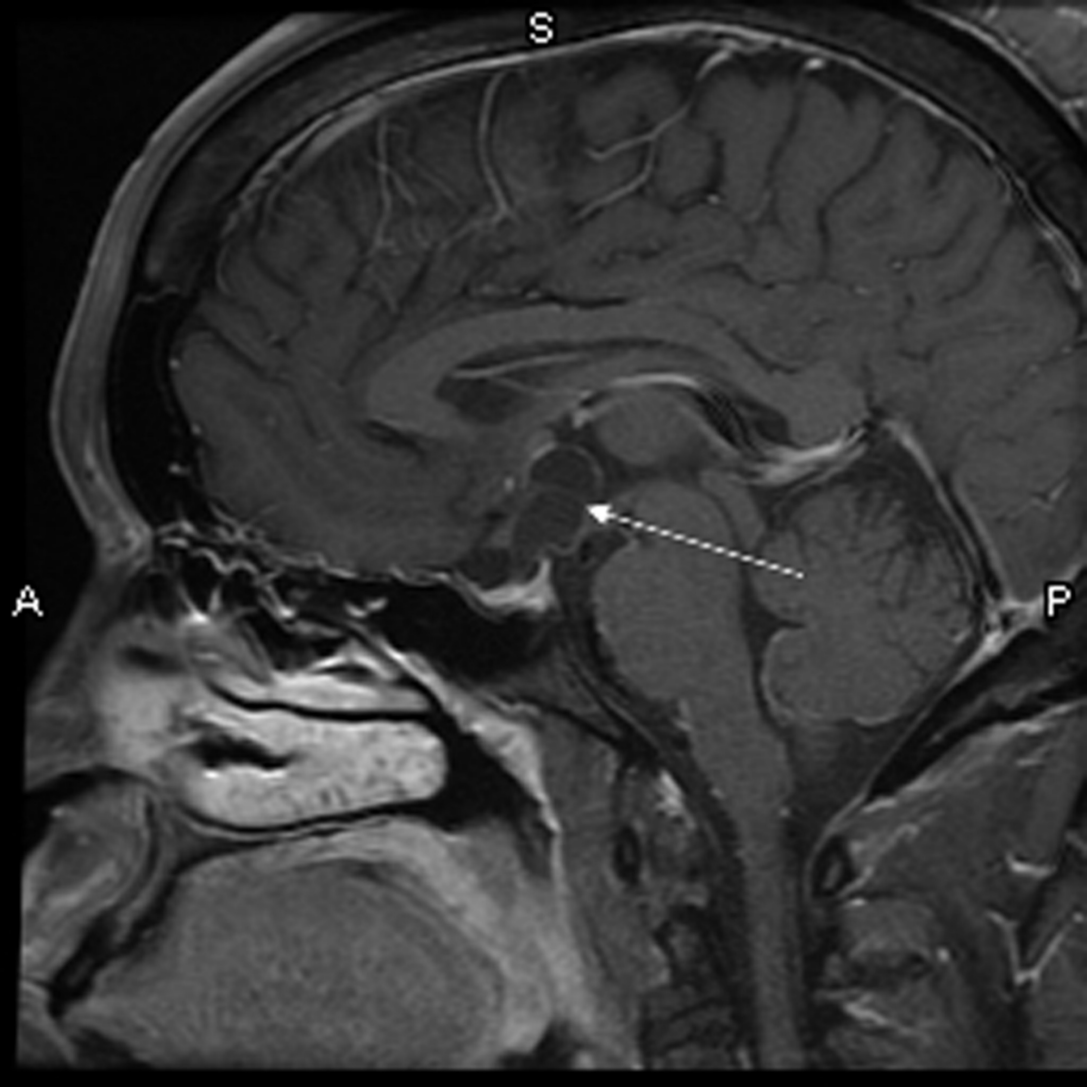
Sagittal T1 with contrast Preoperative brain MRI showing a multi-locular suprasellar cyst (solid white arrow) measuring approximately 1.6 x 1.8 x 2.4 cm

**Figure 2:**
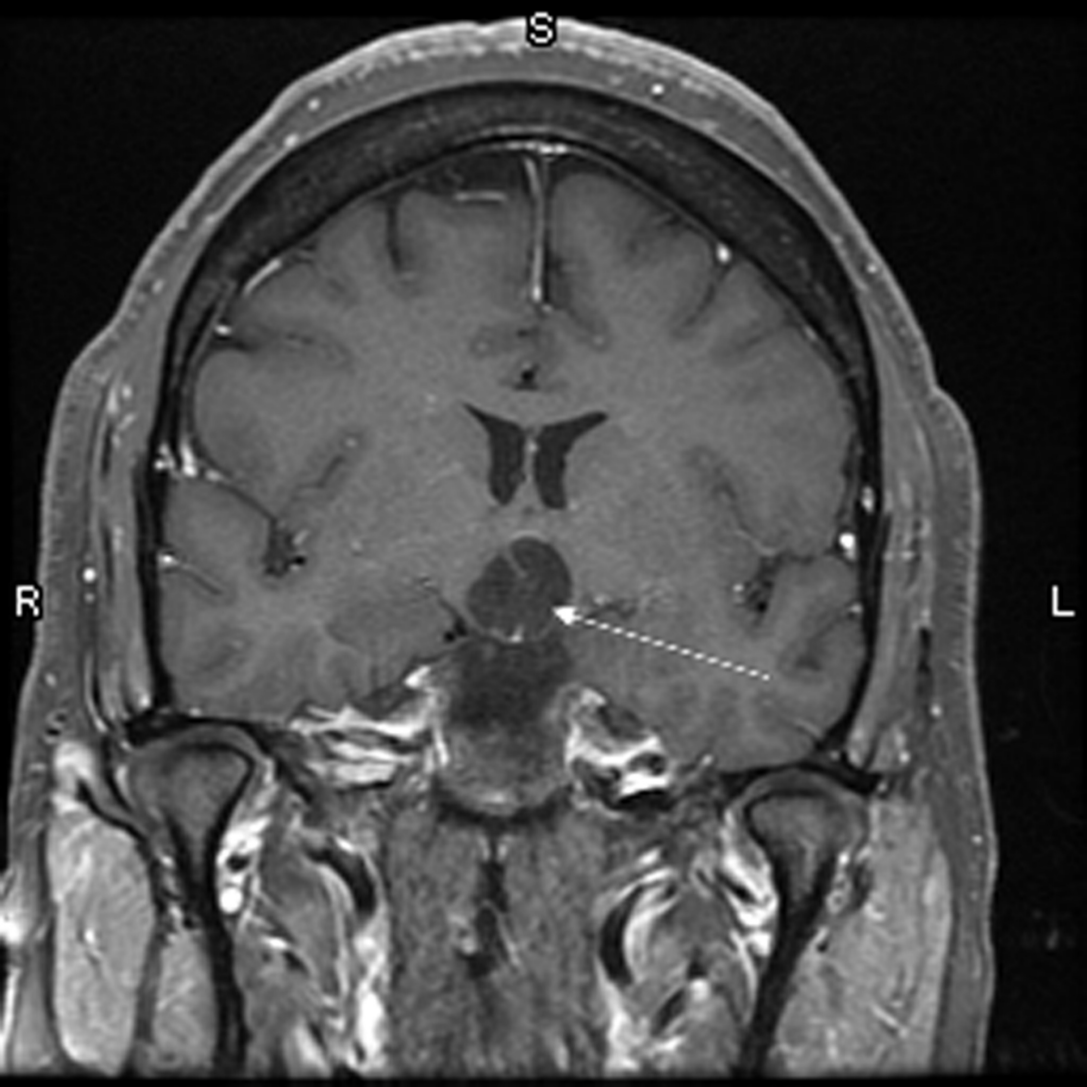
Coronal T1 with contrast Preoperative brain MRI showing a multi-locular suprasellar cyst (solid white arrow) measuring approximately 1.6 x 1.8 x 2.4 cm

**Figure 3:**
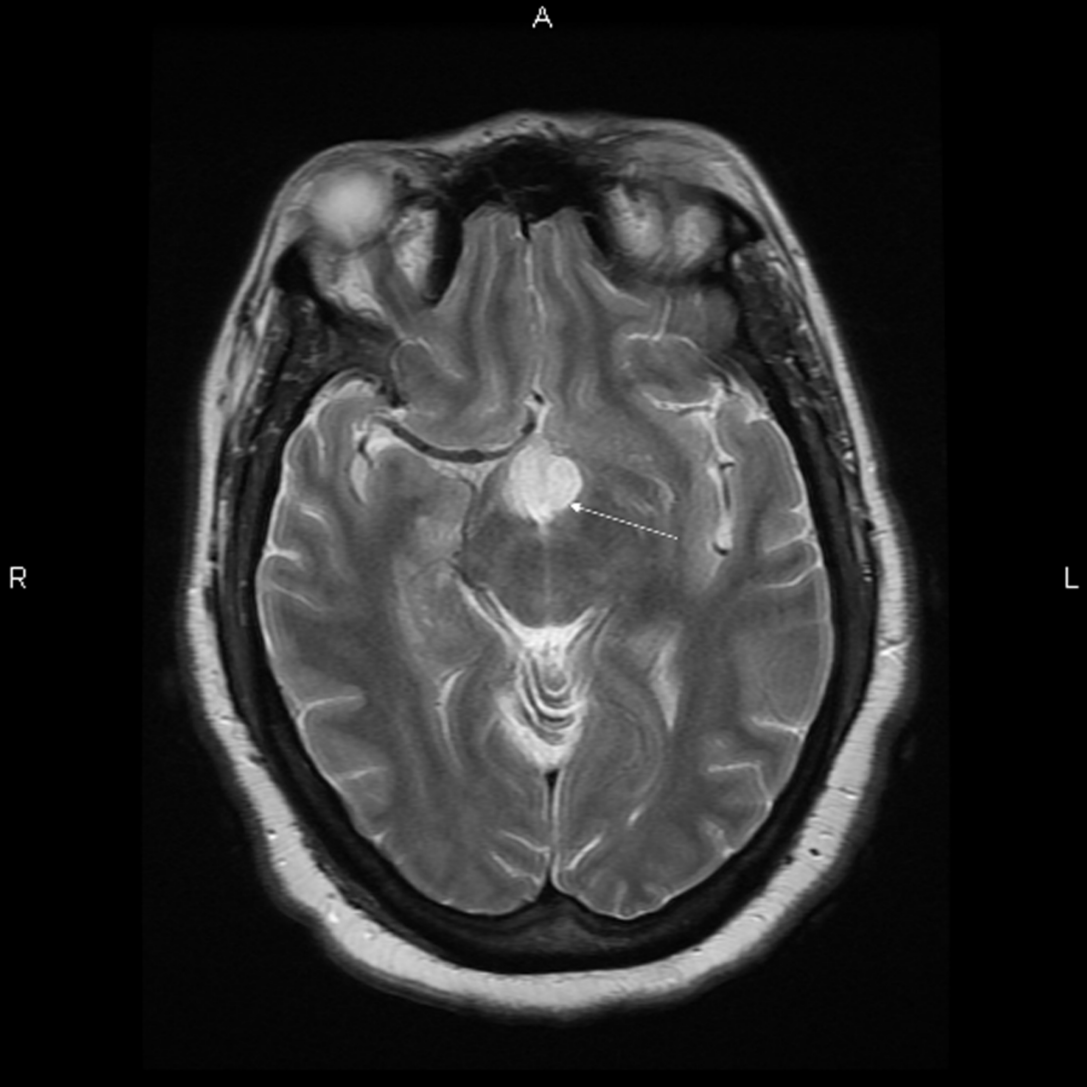
Axial T2 Preoperative brain MRI showing a multi-locular suprasellar cyst (solid white arrow) measuring approximately 1.6 x 1.8 x 2.4 cm

**Figure 4:**
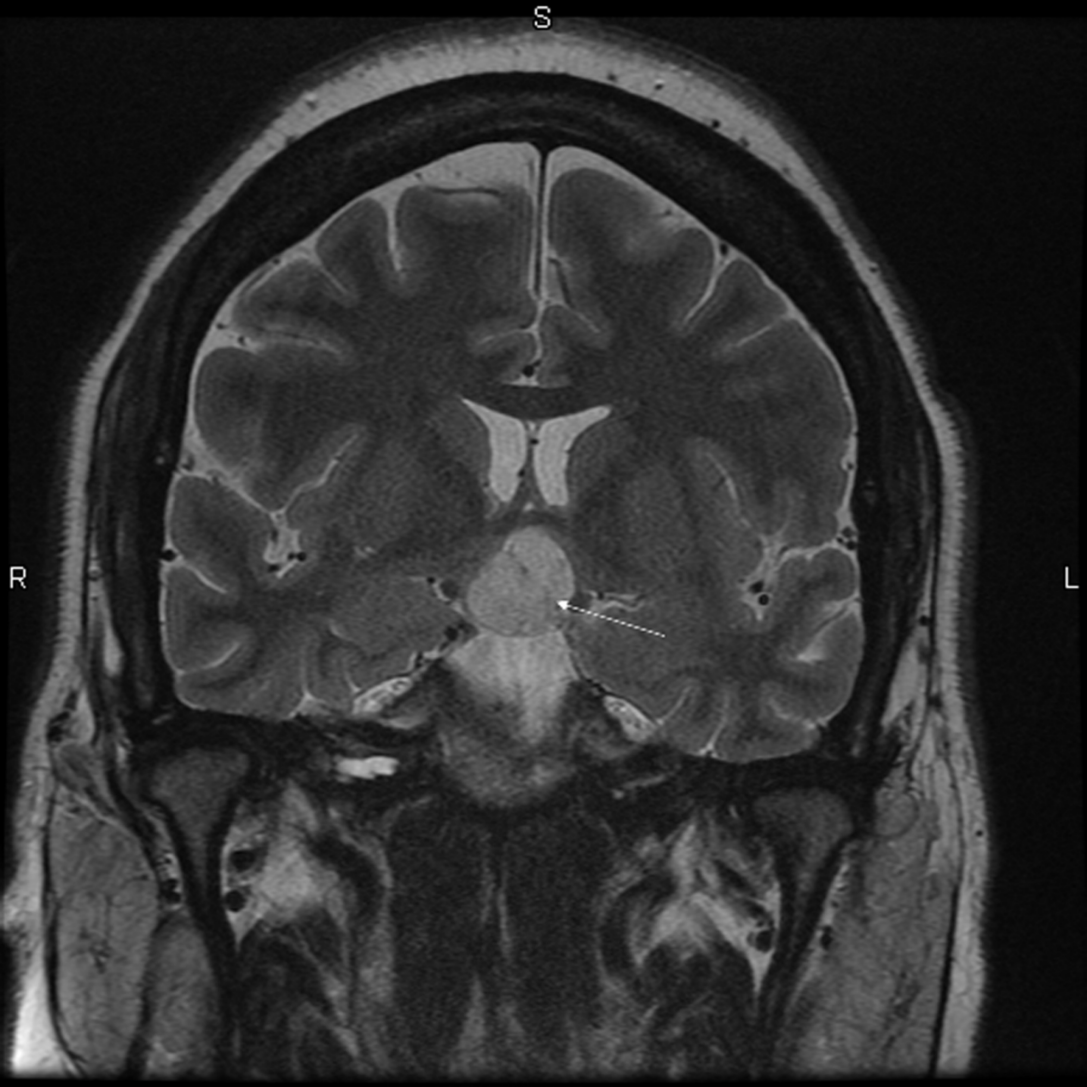
Coronal T2 Preoperative brain MRI showing a multi-locular suprasellar cyst (solid white arrow) measuring approximately 1.6 x 1.8 x 2.4 cm

Brain magnetic resonance imaging (MRI) with and without contrast demonstrated a 1.6 x 1.8 x 2.4 centimeter multi-locular suprasellar cyst within the suprasellar cistern, originally seen on the outside computed tomography (CT) scan, with enhancement along the left lateral and superior walls of the cyst (Figures 1-4).

A leftward deviation of the pituitary stalk and minimal spreading of the optic nerves was noted as well, given the location anterior to the optic chiasm. The pituitary gland itself, the optic chiasm, and the cavernous sinuses were grossly normal. Prior to surgery the patient also underwent CT angiography (CTA) of the head and neck due to a protrusion found on the skull. The scan revealed a bony overgrowth of the external occipital protuberance as well as a two-centimeter cystic lesion in an area of the suprasellar cistern and anterior third ventricle. Of note, the CTA also showed normal intracranial arterial and venous anatomy and the absence of any aneurysms or malformations. At this time, the differential diagnosis presented by neuroradiology included Rathke cleft cyst, neuroepithelial cyst and craniopharyngioma.

Operative and postoperative course

The patient was counseled on the risks and benefits of the operation, and consented to frameless stereotactic endoscopic endonasal extended transsphenoidal resection of her lesion with a lumbar drain and fat graft. Given the presence of hemorrhagic papilledema, the patient consented to evaluation of opening pressure via a lumbar drain given concern for pseudotumor cerebri and increased intracranial pressure. After a standard transsphenoidal opening was prepared by Otolaryngology, we proceeded to mobilize and remove the cyst from the pituitary stalk via a vertical infundibulotomy. A portion of the cyst wall was adherent to the hypothalamus bilaterally and was left in place. The patient was then transferred to the post-operative care unit in stable condition. No intraoperative complications were noted, and the patient was transferred to the neurological intensive care unit (ICU) for further post-operative management.

Postoperatively the patient’s labs and urine output were immediately suggestive of clinical diabetes insipidus (DI). The patient had increased urine output and was started on intravenous fluid replacement as well as desmopressin prior to ICU transfer. On neurological exam, her left maxillary and mandibular trigeminal nerve dermatomes had decreased sensation to light touch and transient exotropia of the left eye was noted, as was the disconjugate gaze. Improvement in disconjugate gaze was noted on post-operative day two, and was only apparent upon opening eyes from rest. The patient’s sodium levels continued to fluctuate and some bleeding from the left nostril as well as coughing up clots of blood was noted four days after the procedure.

**Figure 5:**
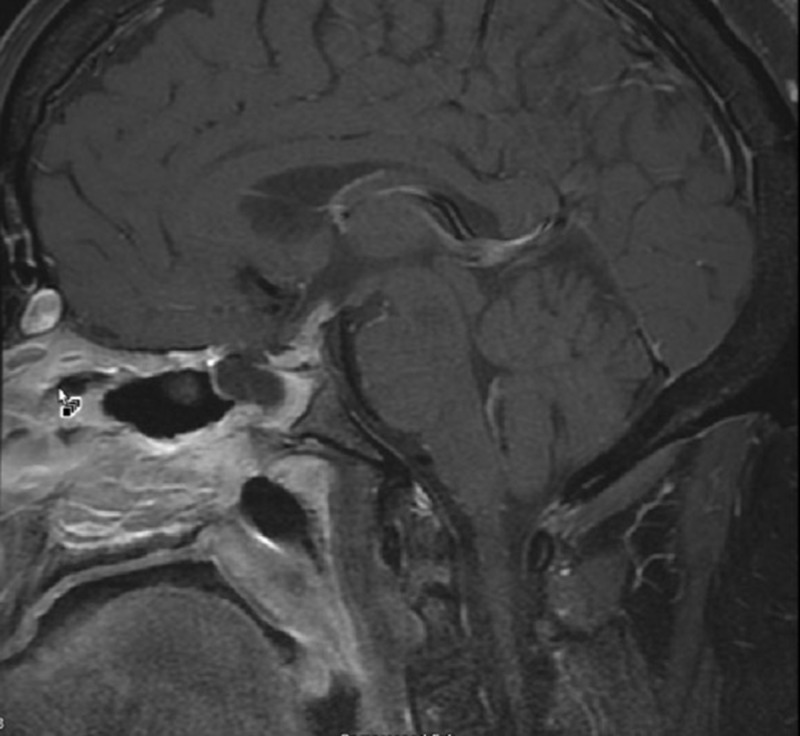
Sagittal T1 (Post-operative) Post-operative brain MRI approximately 8 weeks after surgical resection of the lesion.

**Figure 6:**
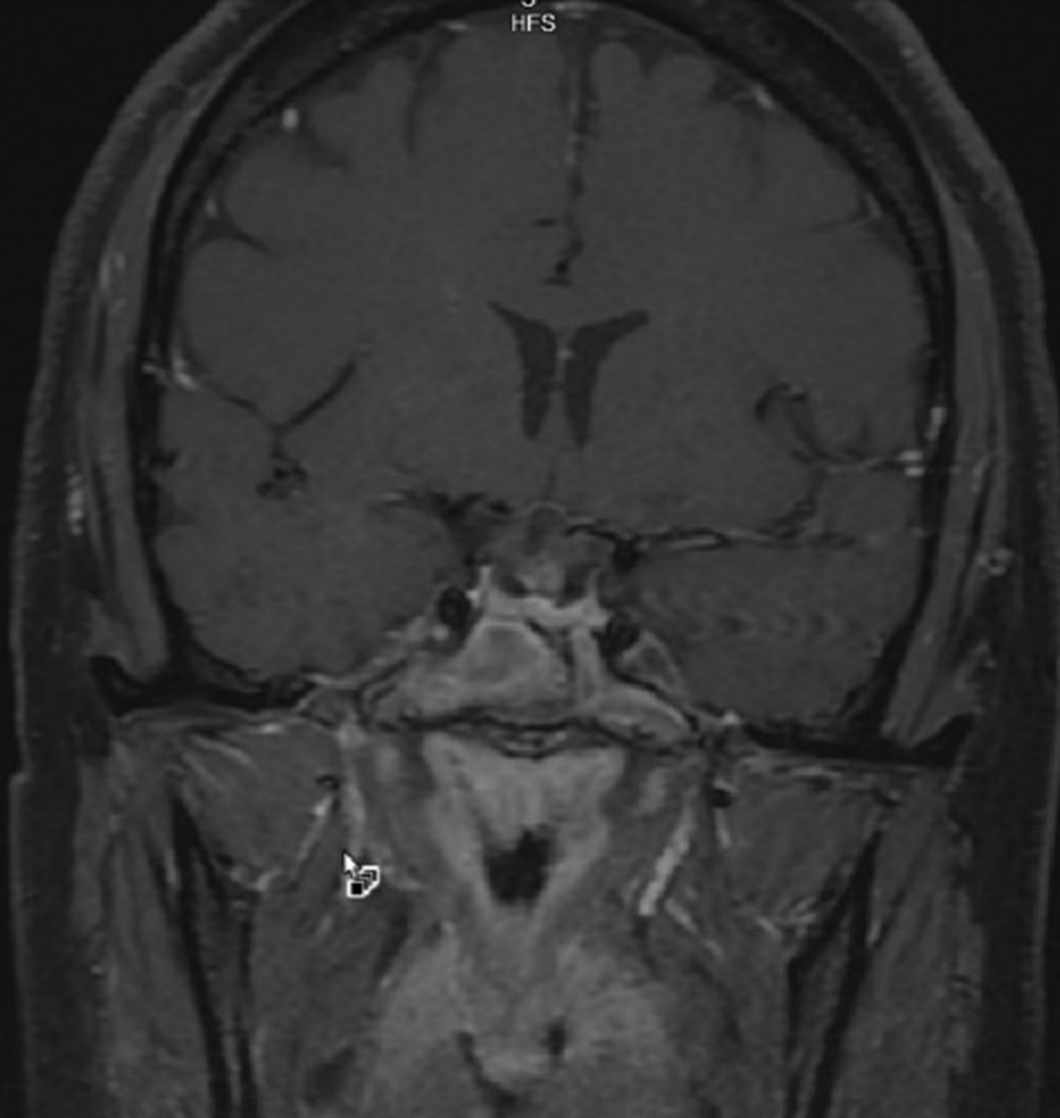
Coronal T1 (Post-operative) Post-operative brain MRI approximately 8 weeks after surgical resection of the lesion.

**Figure 7:**
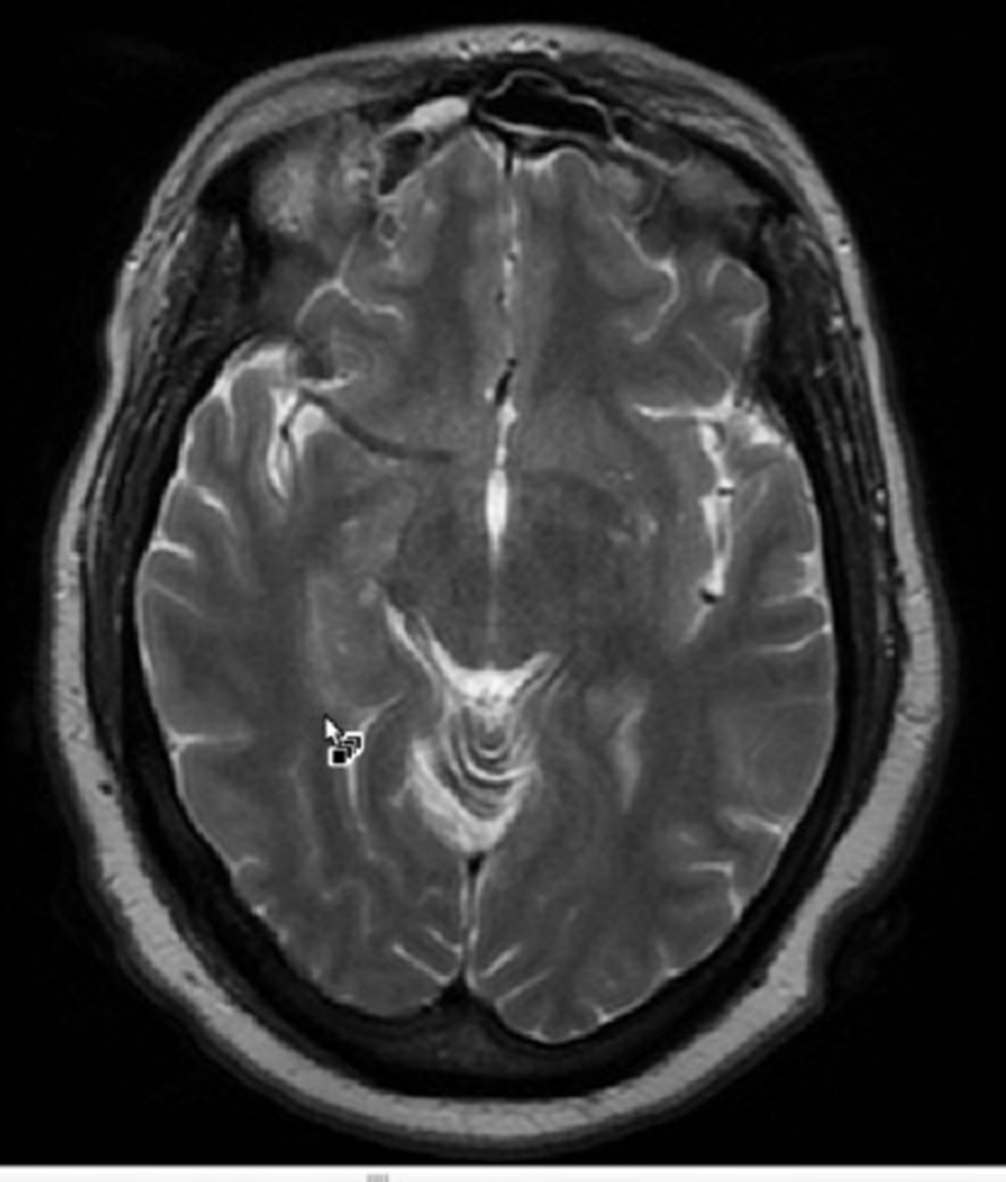
Axial T2 (Post-operative)

**Figure 8:**
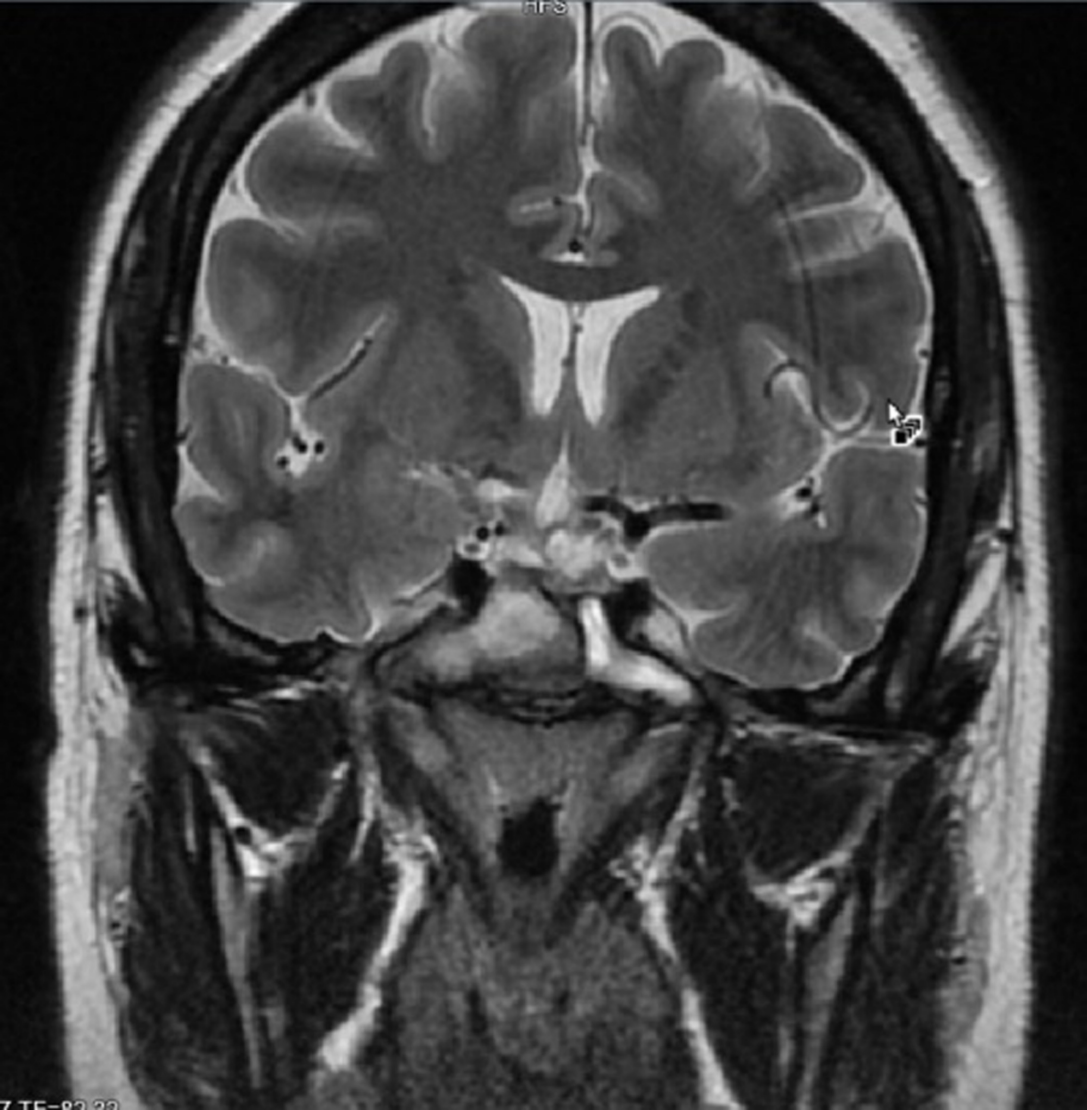
Coronal T2 (Post-operative)

By post-operative day seven the DI resolved and the patient discharged. Given the continued presence of bilateral hemorrhagic papilledema on exam it was recommended at the time that the patient continue treatment with acetazolamide 500 milligrams by mouth twice daily for pseudotumor cerebri. A post-operative brain MRI performed at this time showed resection of the suprasellar lesion (Figures 5-8).

While her DI resolved, the patient continues on hormone replacement for panhypopituitarism.

## Discussion

**Figure 9:**
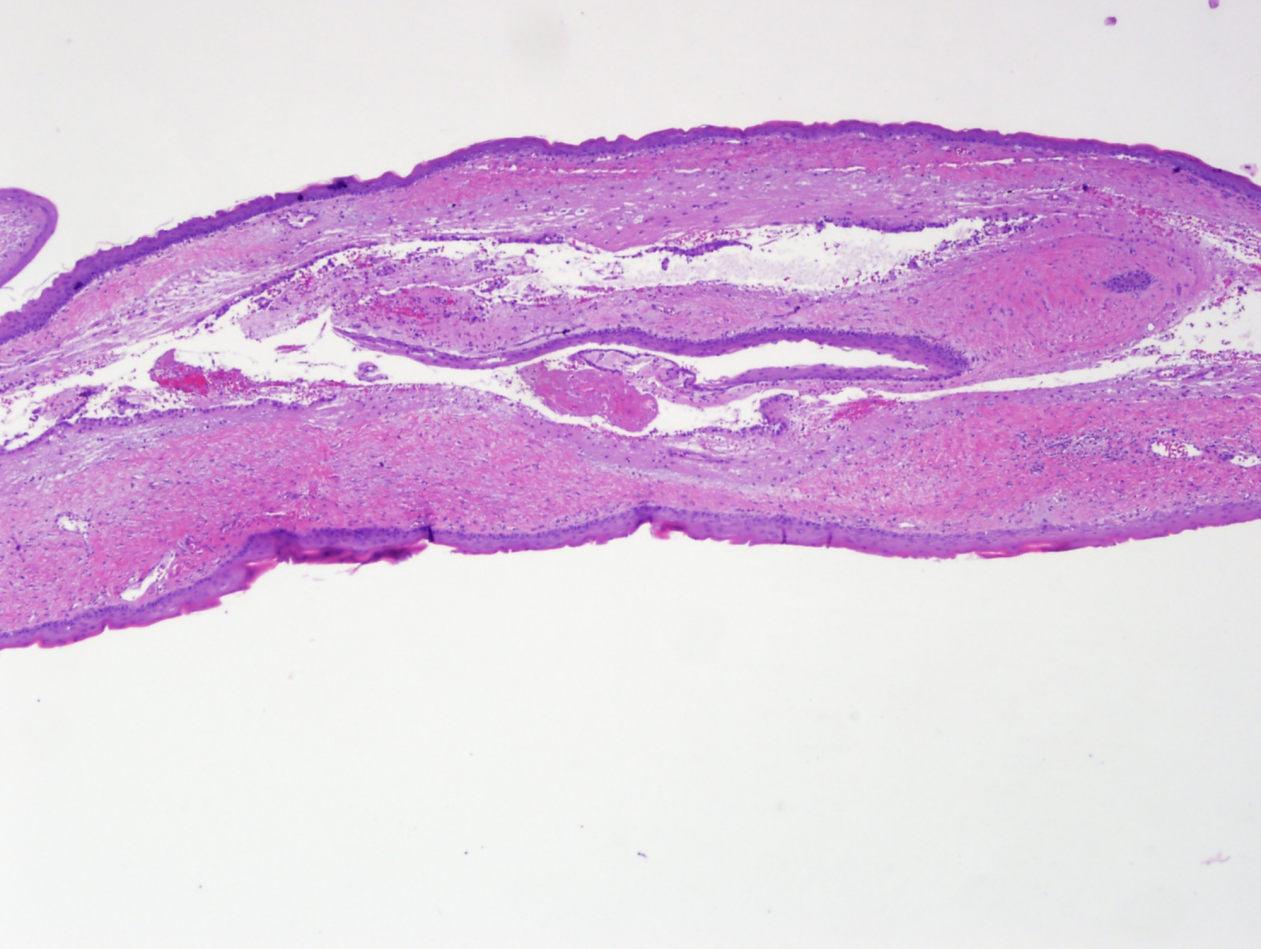
Epithelial-lined cyst (H&E stain, 4x)

**Figure 10:**
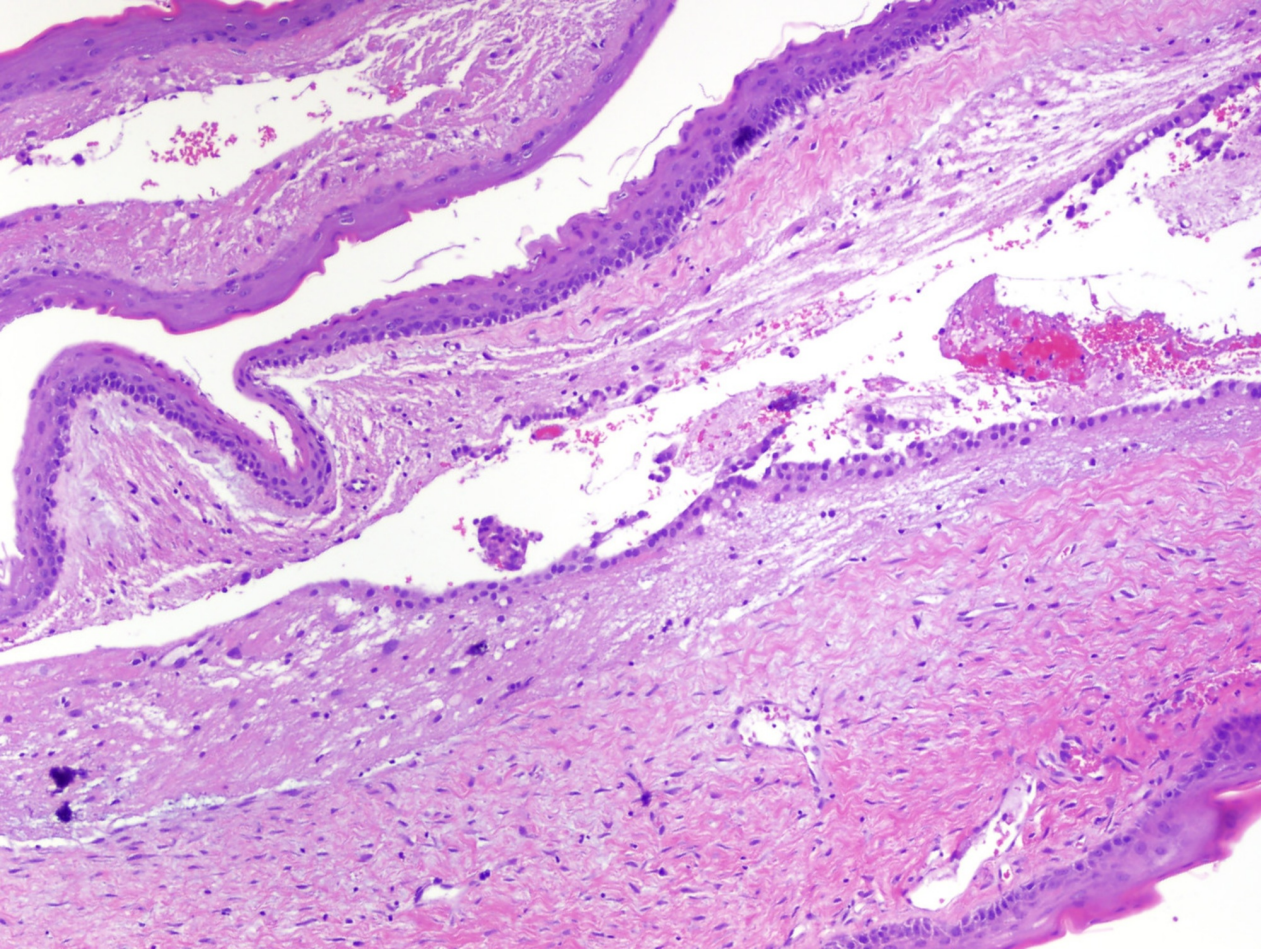
Cyst lining with keratinizing stratified squamous epithelium (arrowhead H&E stain, 10x)

The incomplete separation of the neural and epidermal ectoderm allows for epiblast inclusion in the neural tube, which typically closes during this gestational period [3]. The differential diagnosis for a lesion in this region, especially one presenting within the infundibulum, includes epidermoid cyst, craniopharyngioma, and Rathke’s cleft cyst [2]. Our patient’s cyst was lined by keratinizing stratified squamous epithelium (Figure 9, Figure 10). 

**Figure 11:**
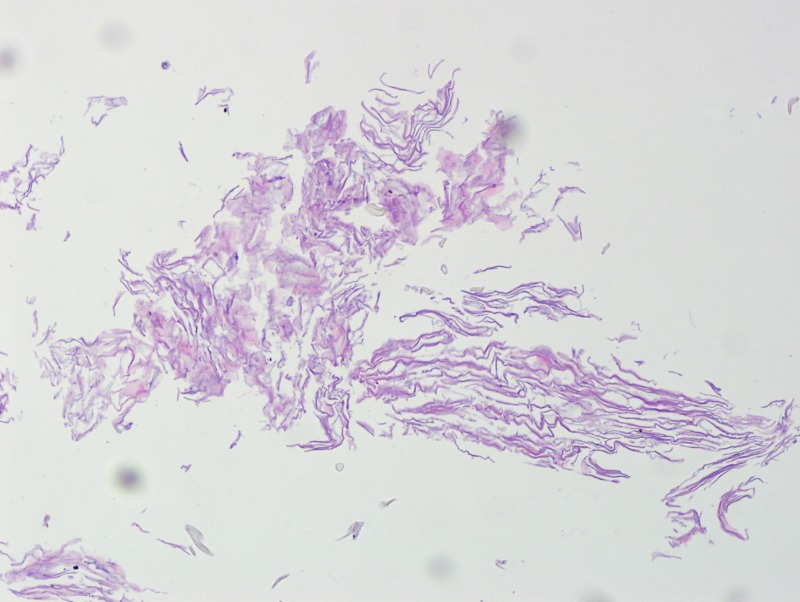
Cyst contents consisting of dry, flaky keratin debris (H&E stain, 10x)

**Figure 12:**
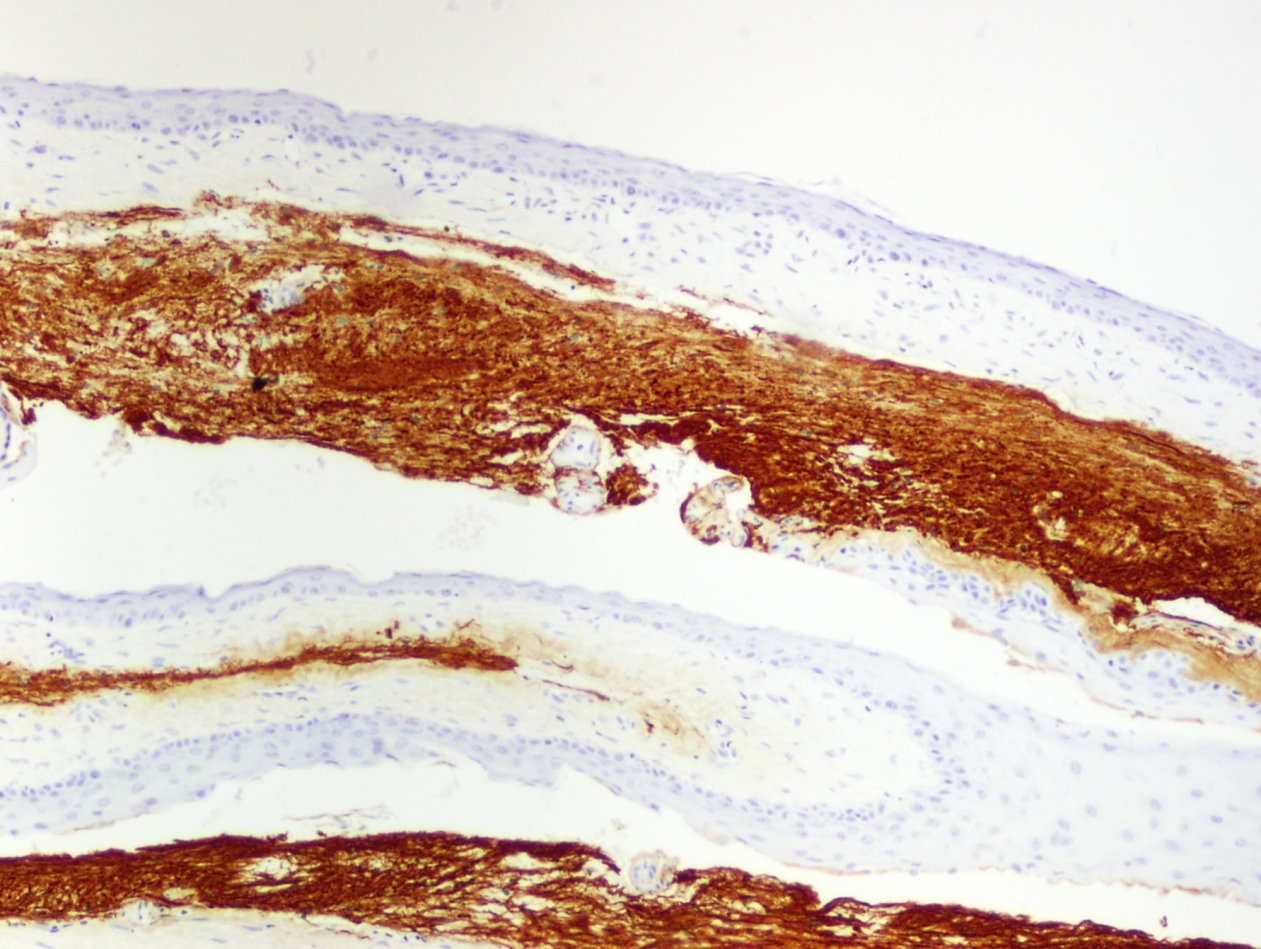
Immunohistochemical stain for glial fibrillary acidic protein (GFAP) highlighting glial tissue opposing the cyst wall (10x)

Additionally, the cyst consisted of dry, flaky keratin debris (Figure 11) and immunohistochemical stain glial fibrillary acidic protein (GFAP) positive (Figure 12) tissue adjacent to the lesion, illustrating the epidermoid characteristics of the cyst and the adjacent glial tissue.

Of note, a minute portion of neighboring epithelium on the pathology sample was positive for epithelial membrane antigen (EMA), which suggests a Rathke’s cleft cyst. Given the common embryological origin of the hypothalamic-pituitary axis, the pathology of several of these lesions are similar and often overlaps. For example, craniopharyngiomas most commonly stem from the infundibulum and are composed of squamous epithelium; however, nearly 60% of these tumors have calcification present [5, 15]. In contrast, epidermoid cysts are filled with keratin debris, lipid, and water and appear hypodense on CT [6, 15]. While our patient’s neoplasm had a detached fragment that was calcified, these tumors are often present in the pediatric population and present with endocrine symptoms in addition to vision loss [5]. The hypodensity of epidermoid lesions is characteristic of these tumors, the lack of enhancement attributed to their low vascularity. On diffusion-weighted imaging, epidermoid cysts appear hyperintense in comparison to arachnoid cysts and other tumors commonly confused with these cysts [7, 15].

Epidermoid cysts are slow-growing tumors and their linear growth rate accounts for the slow onset of symptoms as the cyst expands. These cysts are believed to develop during the embryonal period of development, more specifically the third to fifth weeks of gestation, with displacement of dorsal ectodermal cells normally residing in the midline [3]. With regard to clinical presentation, endocrine silent pituitary tumors account for 15%-20% of intracranial neoplasms. While most brain tumors present with headache, the headache associated with epidermoid tumors has not demonstrated correlation with the size of the tumor [8]. Current teaching suggests that the stretch of the sellar region and medial wall of the cavernous sinus are influenced by intrasellar hypertension. In addition, visual field defects are common once the tumor begins to expand in the suprasellar region, coming into contact with the optic chiasm and optic nerves, but are not necessarily symmetrical [8]. Most commonly, epidermoid tumors are clinically silent until cranial nerve involvement or mass effect produces noticeable symptoms [3]. Symptoms may present differently depending on if the stalk is being directly compressed or if the stalk itself is disrupted by the lesion. Hormonal changes, such as hyperprolactinemia, are a result of compression of the pituitary stalk whereas dysfunction of the stalk itself may be a result of a tumor growing within the infundibulum [2]. It is possible that these may occur simultaneously within the same patient.

Surgical resection is the standard of treatment for epidermoid cysts, with gross total resection including the cyst wall necessary to prevent recurrence. Epidermoid cysts are rarely completely resected and the degree of resection obtained is limited by adherence to nearby structures [9]. This adherence is believed to originate from an inflammatory reaction that occurs when contents of the cyst spill from tears in the capsule into the subarachnoid space; the reaction that occurs between the spilled content and the nearby structures and vasculature provides for local adhesions [7]. Firm adhesion of the cyst to the underlying pia matter or leptomeninges around cranial nerves are examples of situations in which gross versus subtotal resection would have to be weighed [3]. The clinical decision to reach for total resection is surgeon and patient dependent; the risks and benefits of operating near key vascular structures must be included in the pre-operative discussion, with expectations should complications occur clearly outlined before surgery. Should subtotal resection be the best option for a patient, it is important to note that should further surgery be required for recurrence, the likelihood of adherence to nearby vasculature would provide for a more challenging operation [10].

Of note, Samii et al note that only 50%-80% of patients who undergo surgical resection for intracranial epidermoid cysts obtain complete removal [11]. The complications that can arise with subtotal resection are severe, with 40% of patients acquiring chemical meningitis due to the spillage of cyst contents [7]. The authors of the study listed the following as mitigating factors for the development of chemical meningitis: careful dissection of the cyst to avoid rupture, perioperative steroids, removal of the epithelial cyst lining, and surgical site irrigation with hydrocortisone or normal saline [7]. A similar review of adult patients noted that capsule dissection and peri- and post-operative irrigation with dexamethasone and the use of cotton pads around the cyst reduced the development of aseptic meningitis [10].

In addition to chemical meningitis, spillage of the cyst contents can cause a communicating hydrocephalus that may eventually require shunt placement [7]. Transient diabetes insipidus is a common complication of epidermoid resection in the parasellar region, occurring in slightly less than five percent of patients; however, this condition can be either transient or permanent [4, 12]. While DI is more common after the removal of a craniopharyngioma given its origination in the stalk, it is not unreasonable for this to occur with removal of other tumors involving the infundibulum, as seen in our patient [4]. In regard to epidermoid cysts specifically, spontaneous pre-operative and delayed post-operative hemorrhage are unique to intracranial atypical epidermoid cysts [13].

While it is still unclear if the degree of resection is predictive of recurrence, it has been suggested that it is most likely the re-accumulation of cyst contents that causes the appearance of symptoms [6]. As of 2003, the recurrence rate for intracranial epidermoid cysts was stated at 24% [7]. Given that epidermoid tumors are epithelial in origin, there is a risk for malignant transformation into a squamous cell carcinoma. As of 2014, the risk for transformation ranged from six months to 33 years [10]. Characteristics to support the presence of malignant transformation would include rapid growth and expansion of the tumor and or evidence of enhancement after contrast on neuroimaging [3].

The only other reported case of an epidermoid cyst occurring within the infundibulum involved a young female patient who presented with a two-year history of significant endocrine symptoms including amenorrhea, galactorrhea, polyuria, and polydipsia [2]. This patient also reported visual symptoms and headache, as seen in our patient. Similarities between the two cases include gender, tumor type, and anatomical location; however, our patient was nearly ten years senior and reported a more acute time course of one to two months. Both patients received subtotal resection of the tumor given its adherence to the stalk and close proximity to the optic chiasm. On pathological examination, both cysts contained keratin debris, however, our patient had a small region of calcification, suggestive of a Rathke cleft remnant [2]. While both patients fall into the age range for these lesions, 20-60 years of age, neither fits the peak incidence of the fourth decade of life [14]. The similarities and differences between these cases highlight the variety of symptoms and clinical presentations of tumors residing within this region of the brain and the close attention to detail required in diagnosis.

## Conclusions

Epidermoid cysts of the infundibulum are extremely rare, with only one additional case reported in the literature. Given the location of these cysts and their proximity to key neurovascular structures, the surgical approach should be tailored to each individual lesion keeping in mind the surrounding neuroanatomy. As seen in our patient, tumors involving the pituitary stalk are particularly challenging given the high risk for postoperative endocrinopathy, and managing of surrounding structures including the hypothalamus, optic chiasm and vessels within the cavernous sinus. While other cases have presented with lesions intruding into the sellar region, epidermoid growth within the stalk itself is rare and our patient is an excellent example of the neurosurgical management of an epidermoid cyst residing in this specific location.
